# Regulation of Effector and Memory CD8 T Cell Differentiation by IL-2—A Balancing Act

**DOI:** 10.3389/fimmu.2018.02987

**Published:** 2018-12-20

**Authors:** Vandana Kalia, Surojit Sarkar

**Affiliations:** ^1^Division of Hematology and Oncology, Department of Pediatrics, University of Washington School of Medicine, Seattle, WA, United States; ^2^Ben Towne Center for Childhood Cancer Research, Seattle Children's Research Institute, Seattle, WA, United States; ^3^M3D Graduate Program, University of Washington School of Medicine, Seattle, WA, United States; ^4^Department of Pathology, University of Washington School of Medicine, Seattle, WA, United States

**Keywords:** IL-2, CD8 T cell memory, terminal effectors, autocrine, transcription factors, metabolism

## Abstract

Interleukin-2 (IL-2) regulates key aspects of CD8 T cell biology–signaling through distinct pathways IL-2 triggers critical metabolic and transcriptional changes that lead to a spectrum of physiological outcomes such as cell survival, proliferation, and effector differentiation. In addition to driving effector differentiation, IL-2 signals are also critical for formation of long-lived CD8 T cell memory. This review discusses a model of rheostatic control of CD8 T cell effector and memory differentiation by IL-2, wherein the timing, duration, dose, and source of IL-2 signals are considered in fine-tuning the balance of key transcriptional regulators of cell fate.

## Introduction

Interleukin-2 (IL-2)–the first cytokine to be identified and characterized more than three decades ago—has emerged as a pleiotropic player in a variety of seemingly paradoxical immune functions. Originally discovered for its immunoenhancing role of promoting T cell expansion during mitogenic stimulation, IL-2 is also implicated in activation-induced cell death (AICD). Likewise, IL-2 promotes a variety of effector (cytotoxic CD8, T_H1_) T cell responses, yet is indispensable for the development, maintenance and function of regulatory T cells (T_reg_)—the very cells that serve to suppress effector T cell responses. Further adding to the intrigue, even amongst the effector subsets, IL-2 promotes CD8, T_H1_, and T_H2_ effector responses, but suppresses inflammatory T_H17_ responses, and also inhibits the differentiation of follicular helper T (T_FH_) cells required for B cell germinal center reactions in secondary lymphoid organs. Collectively, these findings support the thesis that IL-2 critically regulates the balance of immunostimulatory and immunosuppressive forces during immune responses to foreign antigens as well as self-antigens during homeostasis. While our understanding of the molecular, transcriptional, and metabolic regulation of CD4 T cell differentiation into T_H1_, T_H2_, T_H17_, T_FH_, and T_reg_ subsets by IL-2 is abundant [see previous reviews (1–5)], the IL-2-dependent gene regulatory networks that drive effector and memory CD8 T cell differentiation remain to be fully defined. In this review we will focus on IL-2 regulation of CD8 T cell responses; alongside a summary of current literature in the context of CD4 and CD8 T cells, we will also discuss how this niche area is poised for significant advances owing to newer tools such as conditional ablation of IL-2 production and signaling in key subsets of immune cells in the physiologically relevant setting of immunocompetent hosts.

## Balancing Primary and Secondary CD8 T Cell Immunity

### CD8 T Cell Responses to Acute Infections

A typical CD8 T cell response to primary infection with acute viral or intracellular bacterial pathogens is characterized by three distinct phases—expansion, contraction, and memory. Upon stimulation with cognate antigen in conjunction with costimulatory and inflammatory ligands, naïve cells undergo massive clonal expansion (up to 50,000-fold) and concomitant effector differentiation to generate large numbers of cytotoxic T lymphocytes (CTL), which serve to control the pathogen by migrating to peripheral sites of infection and elaborating cytotoxicity against infected target cells and producing effector cytokines such as IFN-γ and TNF-α (6–11). It is now well-established that the effector CTL pool broadly contains two distinct subsets—(1) short-lived effector cells (SLECs), which are fated to rapidly die after pathogen clearance, and (2) memory precursor effector cells (MPECs) (12–16), which are imprinted with antigen-independent survival capabilities for mediating long-term protection against secondary challenge (17–19). Thus, supporting the concept of memory programming, or imprinting of cardinal memory properties during primary expansion (20–23), several studies have now demonstrated that the balance of MPECs and SLECs can be altered by manipulating the duration of antigen, IL-2 and other inflammatory cytokine signals (14–16, 24). In fact, as discussed later, the heterogeneity of the memory CD8 T cell pool is likely programmed by differential signals accrued during the primary expansion phase. IL-2 signals (paracrine or autocrine) in particular exert crucial roles in effector and memory differentiation and function.

### Regulation of Effector CD8 T Cell Responses by IL-2

Optimal T cell activation with cognate peptide-MHC-I and costimulatory ligands result in IL-2 production and induction of IL-2Rα (CD25) expression, which along with IL-2Rβ (CD122, also used for IL-15 signaling), and IL-2Rγ (CD132, also referred to as common γ-chain as it is shared by other cytokines of the γ-chain family such as IL-4, 7, 9, 21) (5), forms the high affinity heterotrimeric receptor for robust IL-2 signal transduction and clonal expansion and effector differentiation (2). Much of the early work on IL-2 regulation of T cell responses relied on reductionist *in vitro* studies where amount and duration of TCR and IL-2 stimulation can be tightly controlled. These studies established a critical role for IL-2 as a T cell growth factor in driving cell cycle progression and expansion of CD8 T cells following TCR stimulation (25). Similar conclusions were reached following *in vivo* administration of IL-2, which engendered enhanced effector and memory pools of antigen-specific CD8 T cells (26–29). While these studies demonstrate that CD8 T cell differentiation events are amenable to manipulation by IL-2, physiological relevance of IL-2 in shaping a developing CD8 T cell response was uncovered following the development of *Il2* germline-deleted mice. Studies in IL-2 knockout mice are confounded by Treg deficiency and associated spontaneous lymphoproliferative disease (30, 31). Hence, irreconcilably disparate outcomes of reduced or unaltered expansion and effector differentiation were reported in the context of infections and peptide immunization in IL-2 knockout mice (32–35). Nonetheless, bypassing pleiotropic immune effects in straight IL-2 and IL-2Rα (CD25) knockout mice, subsequent studies engaged the strategy of adoptively transferring IL-2- or IL-2Rα-deficient TCR transgenic CD8 T cells into wild-type recipients. In these studies, enumeration of antigen-specific CD8 T cells in an otherwise wild-type milieu using congenic differences without the need for restimulation, clearly established a requirement for IL-2 signals in driving optimal primary expansion of antigen-specific CD8 T cells in secondary lymphoid as well as non-lymphoid tissues (36, 37). IL-2 promotes effector differentiation through STAT-5-mediated Blimp-1-dependent induction of effector molecules (16, 38–42). In this regard, proinflammatory cytokine signals such as IL-12, IFN-γ, and type-1 interferons (IFN-α/β)—commonly referred to as signal 3 for their role in promoting optimal clonal expansion of effector CD8 T cells—are believed to complement IL-2, possibly non-redundantly (43, 44). Such collaboration, particularly between IL-12 and IL-2 has been recently shown to be important for optimal expression of transcription factors T-bet and Blimp-1, which synergize to drive a terminal effector differentiation program in CD8 T cells (45).

### Regulation of Memory CD8 T Cell Responses by IL-2

In addition to promoting CD8 T cell expansion and effector differentiation, IL-2 signals are also necessary for memory responses. IL-2Rα upregulation early after TCR stimulation is critical for formation of memory cells with robust secondary expansion capability (46, 47). Subsequent correlations of the duration of IL-2Rα expression with final memory outcome in a physiologically relevant setting—where the natural course of CD8 T cell response was not disturbed—revealed that rapid downregulation of IL-2Rα is equally important for memory development (16). Fate-tracking analyses showed that following an initial burst of IL-2 signals through IL-2Rα, curtailed expression of IL-2Rα and diminished IL-2 signaling is associated with memory fate, whereas prolonged expression of IL-2Rα and stronger IL-2 signaling drives terminal effector differentiation (16). Stronger IL-2 stimulation (100 U/ml) during *in vitro* priming also drives terminal differentiation compared to weaker signals (10 U/ml) (41). Similar findings have been reported in the DC-peptide immunization models as well as during murine infection with Lymphocytic choriomeningitis virus (LCMV), Listeria monocytogenes (LM), Vaccinia virus (VV), and Vesicular stomatitis virus (VSV) (16, 48). Moreover, constitutive activation of STAT-5 (key signal transducer of common γ-chain cytokines) also causes terminal differentiation (49). Consistent with the pro-proliferative role of IL-2, terminally differentiated effector CD8 T cells (SLECs) that express IL-2Rα for longer duration during an acute infection expand more than their memory-fated counterparts (MPECs) that downregulate the expression of IL-2Rα earlier (15, 16, 50–52). Together, these findings support the notion that metered IL-2 signals are required for optimal protective immunity and present a model of rheostatic control of CD8 T cell fates by IL-2 during acute infections.

All memory cells that survive after clearance of a primary infection are not created equal. Protective CD8 T cell immunity, as we understand it today, consists of collaborative defense against secondary challenge through concerted actions by a complex mixture of memory cells with distinct phenotypes, location, migratory properties, polyfunctionality, antigen-independent longevity, and potential for mounting rapid and robust clonal expansion and effector functions upon secondary challenge (44). As is expected from a spectrum of effector CTLs—that develop in response to varying doses and durations of antigen perceived in a variety of immune contexts, such as dose and duration of cytokines (e.g., IL-2, IFN-I, IL-12, IL-21, TGFβ, etc.), costimulatory signals, CD4 T cell interactions—a veritable spectrum of memory cells exist in a host after antigen clearance. At the risk of oversimplifying the CD8 T cell memory complexity, one can arguably categorize memory cells broadly into two major subsets—lymphoid or central memory (T_CM_), and non-lymphoid memory, which is further distinguished into tissue-resident memory (T_RM_), and migratory memory. Defined by their location, central memory cells largely recirculate through secondary lymphoid organs; tissue-resident memory (T_RM_) cells—true to their name—set up permanent residence at front-lines of pathogen exposure; whereas migratory memory cells comprise a heterogeneous population that is capable of recirculation to peripheral tissues, and may be further distinguished by intravascular staining methodology into the CX3CR1^hi^ effector memory subset (T_EM_) which does not enter extravascular space, and the less differentiated CX3CR1^int^ memory subset capable of migration into extravascular spaces (53, 54). T_RM_ cells serve effectively as the first line of defense against infections by virtue of their key properties of location at barrier sites and rapid elaboration of effector functions (cytotoxicity against infected target cells and effector cytokine production). Consistent with their ability to recirculate through peripheral tissues, T_EM_ cells retain higher expression of effector molecules, and are believed to aid T_RM_ cells in protecting against secondary challenge along with the extravascular migratory memory cells. In contrast, T_CM_ cells largely downregulate their effector program after antigen clearance, but are capable of rapid upregulation of the effector program upon antigenic rechallenge, also have superior polyfunctionality (ability to coproduce multiple cytokines such as IL-2, IFN-γ, and TNF-α), and expand more vigorously to aid the T_RM_ and migratory cells during secondary challenge.

Developmentally, fate-tracking experiments show that effector CD8 T cells that rapidly downregulate IL-2Rα largely give rise to central memory and effector memory cells. In comparison, effector CTLs, with prolonged IL-2Rα expression, largely give rise to terminal effector and effector memory fates; and curtailed stimulation of these cells by adoptive transfer into infection-controlled recipients (removal of antigen, IL-2, and all other infection-related signals) results in less terminal differentiation, as evidenced by increased proportions of effector memory cells compared to short-lived effector cells. These observations are consistent with a role for increasing IL-2 in driving effector CD8 T cells progressively toward terminal differentiation. It is believed that T_RM_ cells arise from relatively less differentiated memory precursors, which first seed the peripheral sites such as skin and small intestines (55, 56). *In situ*, the precursors receive microenvironment-specific developmental cues that drive the expression of unique chemokine receptors, integrins, and transcription factors for T_RM_ cell tissue residency and local protection (56–61). Within the tissue, the transforming growth factor β (TGF-β) exerts a critical role in directing the T_RM_ differentiation program in concert with other tissue-specific signals (55, 56, 62, 63). While CD8 T_RM_ cells capable of IL-2 production have been recently reported in skin and liver (64, 65), and IL-2 signals have been shown to be important for maintenance of allergic T_H2_-type cells in the lungs (66), murine studies directed at understanding whether early IL-2 signals are necessary for T_RM_ seeding of tissue sites, whether prolonged IL-2 signals compromise T_RM_ cells, and how TGF-β signals and other tissue-specific factors work in conjunction with IL-2 signals (synergistically or antagonistically) to drive the differentiation, maintenance, and recall function of T_RM_ cells within the local sites remains to be fully explored. Likewise, whether similar rules of progressive terminal differentiation with increasing IL-2 signals are also active in situations of chronic antigen stimulation—as occurs during persistent viral infections and cancers—remains to be defined.

## Autocrine and Paracrine Programming of T Cell Fates

During thymic development, T cell-derived IL-2 is critical for development of Treg cells. (67). During homeostasis, IL-2 is largely produced by CD25^int^ and CD25^lo^ CD4 T cells (activated by self-peptide and foreign peptide MHC-II complexes on DCs) (68), the regulatory T_R1_ subset in peyer's patches that also produces IL-10 and IFN-γ (69), and to some extent by NK, NKT, and CD8 T cells [evaluated by mRNA (68)]. Recent studies, involving IL-2 ablation in defined immune cells, have shown that T cell-derived IL-2 is critical for maintaining numbers and regulatory function of Treg cells in most secondary lymphoid organs, with the exception of mesenteric lymph nodes where DC-derived IL-2 was also observed to be important (67). During an immune response, activated CD4 T cells produce copious amounts of IL-2 (2), with other IL-2 producers being CD8 T cells (70), DCs (71), NKT cells (72), and mast cells (73). There is evidence that IL-2 may be transpresented by CD25 expressing DCs (74) to deliver high affinity IL-2 signals to CD8 T cells that lack CD25 expression and only express the intermediate affinity β/γ IL-2 receptor heterodimer—analogously to IL-15 transpresentation—thus, suggesting that IL-2 may be delivered in a context-specific manner *in vivo* depending on the nature and activation status of antigen-presenting DCs.

With highest levels in secondary lymphoid organs, IL-2 is believed to act in an autocrine or paracrine manner to support effector and memory CD8 T cell differentiation. We have previously shown that memory-fated effector CD8 T cells selectively retain the ability for robust IL-2 production in response to antigenic stimulation compared to their terminally differentiated effector counterparts (8, 15, 16, 44). Likewise, polyfunctionality—the capacity for potent IL-2 production along with other effector cytokines such as IFN-γ and TNF-α in response to antigenic restimulation –is a hallmark property of lymphoid central memory CD8 T cells. Querying the functional relevance of autocrine IL-2 production by memory-fated CD8 T cells, studies involving ablation of *Il2* in a fraction of antigen-specific CD8 T cells during attenuated LM immunization (75) as well as acute LCMV infection (unpublished observations), demonstrate that the IL-2 needed for development of robust memory CD8 T cells capable of optimal secondary expansion is largely autochthonous. Since CD4 T cells are the major producers of IL-2, it was long presumed that IL-2 serves as the mode of CD4 help for development of protective memory CD8 T cells capable of robust secondary expansion. Thus, largely dismissing CD4 T cell-derived paracrine IL-2 as a mode of help, it is now proposed that CD4 T cells license DCs through the CD40-CD40L axis to induce memory-fated CD8 T cells to produce IL-2 (75). Autocrine IL-2 production through CD27 signals has also been shown to sustain survival of antigen-specific CD8 T cells in virus-infected non-lymphoid tissues (76, 77).

We further employed novel conditional IL-2 gene-deleted mice (78) to investigate whether autocrine IL-2 signals are specifically required during the programming phase of primary responses, or during secondary expansion (unpublished observations). Ablation of *Il2* in memory CD8 T cell immediately prior to rechallenge did not result in compromised secondary expansion, but ablation prior to primary infection resulted in defective recall responses. These data suggest that autocrine IL-2 signals during primary CD8 T cell expansion are required to institute a program of optimal secondary expansion. In the context of CD4 help to CD8 T cells, these instructive autocrine IL-2 signals are believed to in part promote the expression of a transcriptional corepressor, Nab2 for blocking TRAIL-mediated apoptosis during secondary expansion (79, 80). Defects in protective CD8 T cell immunity associated with IL-2Rα ablation are rescued by a strong bolus of exogenous IL-2 during primary expansion (47), further supporting the idea that IL-2 exerts an early instructive role. Whether secondary expansion defect associated with lack of autochthonous IL-2 maybe similarly rescued by excessive paracrine IL-2 signals remains unknown. Alternatively, it is possible that there are fundamental differences (quantitative and/or qualitative) between autocrine and paracrine IL-2 signals. In the case of CD4 T cells, autocrine IL-2 production in response to cognate antigen and CD70 signals during late stages of influenza A virus infection has been shown to be critical for upregulation of IL-7Rα (CD127) and survival into memory phase (81). More recently, T_FH_ and T_H1_ fates have been linked to autocrine and paracrine IL-2 signals, respectively, with different gene expression programs being triggered for lineage determination in IL-2-producing and non-producing CD4 T cells (82). While CD8 T cells that receive differential strength or duration of IL-2 signals have expectedly unique gene expression programs, it remains to be defined how autocrine and paracrine IL-2 signals impact CD8 T cell gene regulation and metabolism.

## Fine-Tuning The Regulators of T Cell Fates

### Transcriptional and Metabolic Regulation of CD8 T Cell Differentiation

**Figure 1:**
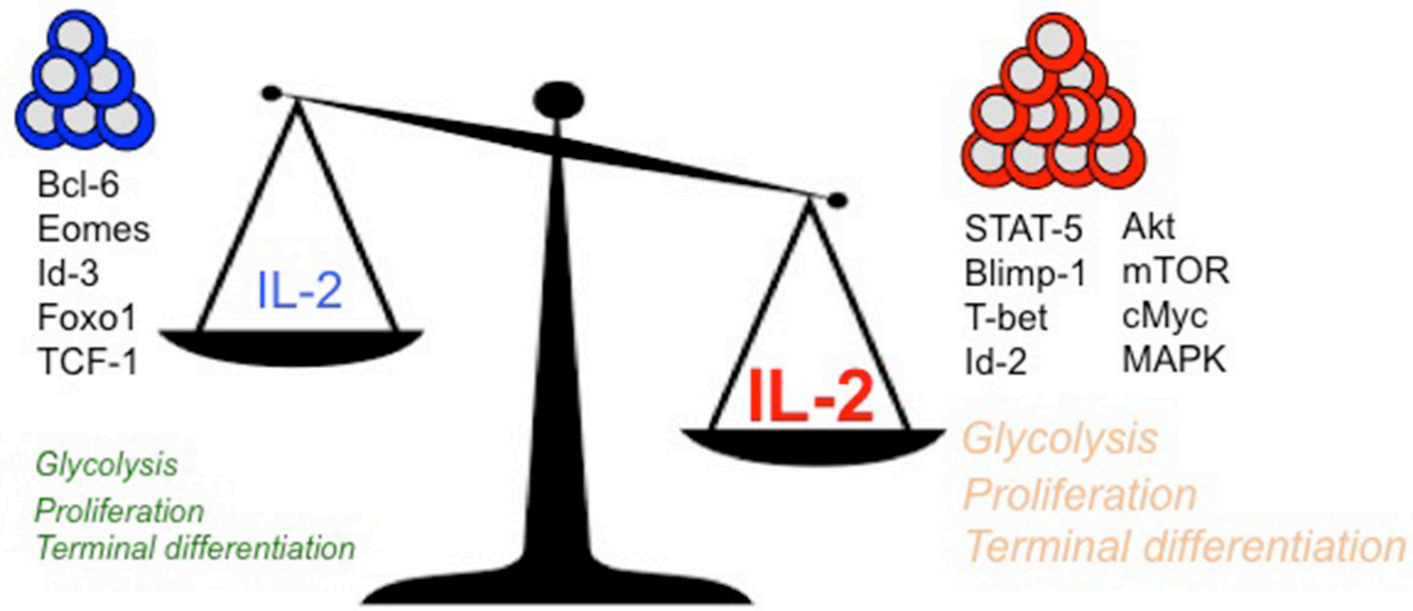
Regulation of key intracellular signaling, transcriptional, and metabolic mediators of terminal effector vs. long-lived polyfunctional memory CD8 T cell fates are presented in the context of differential levels of IL-2 signals.

IL-2 couples T cell expansion and effector differentiation through induction of multiple downstream signaling cascades. Expression of pro-differentiation transcription factors, Blimp-1 (16, 38, 40–42) and Id-2 (83), is largely mediated through STAT-5 activation in response to IL-2 stimulation (2) (Figure 1). Reciprocal suppression by IL-2 of transcriptional factors that promote T cell memory such as Bcl-6 (41, 84–88) (which also represses Blimp-1 expression) is believed to further fix the terminal effector differentiation program (45). IL-2 is believed to regulate the expression of Bcl-6 through activation of Akt, which serves to control the activity of Foxo family transcription factors (89), Activation of Akt also alters the expression of proteins involved in CD8 T cell trafficking such as CD62L, CCR7, and S1P1, so as to promote their migration to peripheral sites of infection and inflammation (90–92). In addition to activation of STAT-5 and Akt, which largely promote effector differentiation, IL-2 links effector differentiation with clonal expansion through activation of MAPK signaling and T cell activation, cell cycle progression and survival programs (89). Sustained expression of cMYC through IL-2 drives proliferation by upregulating cyclins and anti-apoptotic molecule B-cell lymphoma 2 (Bcl-2), and by downregulating p21 (93, 94). In addition to cell cycle regulators, Myc also controls key metabolic aspects of T cell activation and proliferation (95). Myc promotes glycolysis and glutaminolysis through upregulation of key enzymatic and transporter proteins (96, 97). In this regard, mTOR also serves as a primary hub to integrate environmental cues from growth factors such as nutrients and IL-2 to promote glycolysis (94, 98, 99), oxidative phosphorylation and anabolic processes such as protein, lipid, and nucleotide biosynthesis necessary to sustain proliferation (96, 97). How effector and memory CD8 T cell fates are defined *in vivo* through differential metabolic programming by varying IL-2 strength or duration remains to be elucidated.

### A Model for Rheostatic Control of T Cell Fates by IL-2

With diverse outcomes of memory and terminal effector differentiation in CD8 T cells that receive short/weak as opposed to prolonged/strong IL-2 signals (as described earlier), it remains to be determined whether rheostatic control of transcriptional and metabolic regulation occurs. It is plausible that curtailed or weak IL-2 signals drive lower levels of STAT-5, Akt and mTOR activity, thus resulting in lesser proliferation, effector differentiation and trafficking to peripheral sites of infection. In contrast, strong and prolonged IL-2 signals may drive stronger STAT-5, Akt, mTOR and MAPK activity, thus leading to augmented proliferation, effector differentiation and migration to peripheral sites of infection, where the microenvironmental niches further reinforce the terminal differentiation programs through induction of receptors for inflammatory cytokines such as IL-12 (100), and inhibition of IL-7 (101) receptor levels. Notably, a role for Tregs has been implicated in regulating the amount of IL-2 signals to memory-fated CD8 T cells by acting as IL-2 sinks (102) during CD8 T cell expansion. During later stages in the absence of antigen (when IL-2 is limiting) also, Tregs continue to curtail T cell stimulation and proliferation to maintain memory CD8 T cell quiescence through CTLA-4 (103) and IL-10 (104) inhibitory mechanisms and possibly through IL-2 restriction (105). IL-2 is also bound to the extracellular matrix through heparan sulfate moieties (106) to presumably increase local concentrations, thus supporting the notion that strong and prolonged IL-2 signals can be achieved *in vivo*. Effectually, quantal differences in IL-2 signals may lead to differences in signaling thresholds that ultimately result in terminal effector gene expression patterns driven by Blimp-1, T-bet, Id-2, and cMyc, or in memory lineage gene expression patterns characterized by augmented Bcl-6, Eomesodermin and Id-3. Indeed, analogous rheostatic control of CD4 T cell fates by differential levels of IL-2 signaling has been reported in the balance of T_H1_ and T_FH_ fate determination (107, 108) through reciprocal regulation of T-bet and Bcl-6 by mTORC1-dependent control of the glycolysis gene expression program (109).

## Concluding Remarks

Tightly coupled to antigen and costimulation, IL-2 signals follow close suit in T cell activation. In addition to driving expansion and effector differentiation, IL-2 regulates long-term memory outcome as well. Hence, it has been proposed as vaccine adjuvant (110) to augment the size of the memory pool. However, given its rheostatic regulation of terminal effector and memory fates (Figure 1), careful investigation into the dose and duration of IL-2 in a context specific manner is warranted to fine-tune the balance of terminal effector and memory lineages. Hence, based on the clinical need, timely and curtailed IL-2 signals might be exploited to augment memory outcome during vaccination. Alternatively—owing to its ability to induce proliferation and effector differentiation–strong and sustained IL-2 signals might be employed for immunotherapeutic interventions against cancers and chronic infections that rely on activation of a large pool of antigen-specific CD8 T cells. In this quest, IL-2 has gained particular recognition in treating melanomas and renal cell carcinomas (111) by augmenting the tumor-reactive CD8 T cell pool. In the case of gene-modified T cell immunotherapies also—for e.g., when patient T cells are bioengineered to express chimeric antigen receptors or TCRs directed against select tumor antigens–IL-2 is critical for expansion of CAR T cells to sufficient numbers for therapeutic benefit (112). Even in the case of PD-1 checkpoint blockade immunotherapy, IL-2 supplementation has offered combinatorial success with PD-1 blockade in boosting quantitative and functional aspects of exhausted CD8 T cells for enhanced viral control (113). Needless to say, the pleiotropic effects of IL-2 have posed significant hurdles such as off-target side effects of IL-2 administration—e.g., vascular leak syndrome due to activation of endothelial cells, or induction of immune regulation by Tregs. To minimize side effects, novel IL-2 muteins and immune complexes have been developed to selectively target IL-2 to either effector or regulatory T cells (5, 111, 114–116). By enhancing IL-2 binding to the β/γ heterodimer typically expressed on effector CD8 T cells, and thus directing IL-2 away from Tregs—which typically express high levels of IL-2Rα–these immune complexes and muteins provide a means to avoid concomitant induction of Treg suppression observed in case of rIL-2 administration that is counteractive to the desired outcome of effector differentiation. We envisage that detailed molecular dissection of the signal transduction and transcriptional networks downstream of IL-2 signaling *vis a vis* biological outcomes in individual immune cell-types will guide innovative immunomodulatory strategies designed for distinct clinical mandates. Along this concept, manipulations of the Bcl-6-Blimp-1 and CD27-CD70 axes are being considered with the goal of uncoupling effector differentiation effects of IL-2 from expansion effects (89). Beyond the binary terminal effector or memory outcomes conceived thus far, it is enticing to speculate whether rheostatic regulation of MPEC and SLEC differentiation states by controlling IL-2 signals might be exploited to balance the immediate therapeutic benefits and long-term protective outcomes during adoptive T cell therapy and therapeutic cancer vaccines.

## Author Contributions

All authors listed have made a substantial, direct and intellectual contribution to the work, and approved it for publication.

### Conflict of Interest Statement

The authors declare that the research was conducted in the absence of any commercial or financial relationships that could be construed as a potential conflict of interest.
